# Characterizing the Tumor Microenvironment and Its Correlation with cDC1-Related Gene Expression in Gastric Cancer

**DOI:** 10.1155/2024/4468145

**Published:** 2024-07-09

**Authors:** Song-Hee Han, Mi Ha Ju

**Affiliations:** Department of Pathology Dong-A University College of Medicine, Busan, Republic of Korea

## Abstract

**Materials and Methods:**

We analyzed RNA-seq data from the Cancer Genome Atlas (TCGA-STAD) and Gene Expression Omnibus (GEO) datasets, focusing on five cDC1-related genes. The cDC1-related signature was defined and divided into high and low expression groups. We employed gene set variation analysis (GSVA) for oncogenic signaling pathways and conducted comprehensive statistical analyses, including Kaplan–Meier and Cox proportional hazards models.

**Results:**

The high cDC1-related gene signature group was associated with poorer overall and disease-free survival in the TCGA-STAD cohort. Significant differences in CD8+ T cell infiltration and cytotoxic capabilities were observed between high and low CDC1-related signature groups. The study also revealed a strong correlation between CDC1-related signature and increased expression of immune checkpoint proteins and oncogenic pathways, suggesting a complex immunosuppressive tumor microenvironment.

**Conclusions:**

Our findings indicate the potential of the cDC1-related signature as a prognostic marker in GC, offering insights into the tumor–immune interplay. The study underscores the importance of cDC1s in shaping the tumor microenvironment and their influence on patient prognosis in GC. These results may contribute to the development of novel therapeutic strategies targeting the immune microenvironment in GC.

## 1. Introduction

Cancer remains a major global health concern, characterized by its complexity and multifaceted nature [1]. Despite remarkable advancements in treatment modalities, it continues to be a leading cause of mortality worldwide. Central to the pathology of cancer is the ability of tumor cells to effectively evade immune detection and destruction. This evasion is facilitated through various mechanisms, including the secretion of immunosuppressive cytokines, recruitment of immunosuppressive cell types, and upregulation of immune checkpoint molecules (ICPs) [2, 3].

Within the immune landscape, dendritic cells (DCs) play an instrumental role in modulating immune responses, thereby impacting cancer progression [4, 5]. Among them, conventional type 1 dendritic cells (cDC1s) are crucial, responsible for presenting tumor antigens to CD8+ T cells and orchestrating a robust antitumor immune response. Additionally, cDC1s play a vital role in the recruitment and activation of other immune effector cells, including natural killer (NK) cells.

Emerging research suggests a positive correlation between the increased expression of cDC1 transcriptomic markers in various tumors and improved clinical outcomes. For instance, in squamous cell carcinoma of the head and neck, a higher expression of cDC1 transcriptomic signature is associated with better clinical outcomes [6]. Similarly, in breast cancer, enhanced tumor infiltration by cDC1s is linked to favorable prognoses [7]. Moreover, in lung adenocarcinoma, studies have shown a significant decrease in cDC1s under immunosuppressive conditions, as indicated by CyTOF and single-cell RNA analyses, positing cDC1s as key players in the tumor immune environment [8].

In the context of gastric cancer (GC), which ranks as the third most common cancer among Korean men and a leading cause of cancer-related deaths [9], the challenge remains significant despite recent therapeutic advances such as targeted therapies and immune checkpoint inhibitors (ICIs). Thus, the identification of reliable prognostic factors is critical to enhancing patient outcomes. Recent research underscores the potential of immune cells in controlling cancer progression and the effectiveness of immunotherapy in GC [10]. Given this backdrop, our study postulates that cDC1s play a pivotal role in modulating the immune response in GC, thereby influencing the tumor microenvironment and patient prognosis. However, the specific mechanisms through which dendritic cells affect the immune response to GC and its clinical implications remain to be elucidated.

This study aims to evaluate the prognostic significance of cDC1-related genes (CD141(THBD), XCR1, CLEC9A, CADM1, and BTLA) in GC and to unearth the underlying biological mechanisms. By analyzing the expression levels of these genes in GC, using data from TCGA and GEO, and constructing a cDC1 signature divided into high and low groups, we seek to identify key drivers of GC prognosis. Our findings are anticipated to enhance understanding of the biological underpinnings of GC prognosis and contribute to the development of more effective treatment strategies.

## 2. Material and Methods

### 2.1. Data Acquisition and cDC1-Related Gene Expression Analysis

For this investigation, a cohort of 444 patients diagnosed with stomach adenocarcinoma (STAD) and accompanied by RNA sequencing data, along with clinical information, was sourced from The Cancer Genome Atlas (TCGA). To validate the cDC1-related signature, dataset GSE62254 (*n* = 300) was retrieved from the Gene Expression Omnibus (GEO) database. Samples lacking comprehensive data were excluded, resulting in the utilization of 374 TCGA and 300 GSE62254 samples for subsequent analyses. TCGA-STAD served as the training set, while GSE62254 functioned as an independent validation cohort. This study focused on five cDC1-related genes (CD141, XCR1, CLEC9A, CADM1, and BTLA), frequently cited in related literature [11, 12, 13]. The Sangerbox tool supported the bioinformatics analysis. Additional datasets, GSE265254 (*n* = 257), were also obtained from the GEO database, and RNA expression and clinical outcome data pertinent to anti-PD-1 treatment in the PRJEB25780 cohort were acquired from the Tumor Immune Dysfunction and Exclusion (TIDE) database.

Gene expression levels were represented as log-transformed values. The cDC1-related signature (cDC1RS) was defined as the log-transformed geometric mean of expressions for CD141, XCR1, CLEC9A, CADM1, and BTLA. Hyperexpression of a cDC1-related signature was determined if a gene's expression difference ranked in the top 25% across all samples, while hypoexpression was categorized by a ranking in the bottom 25%.

### 2.2. Immune Cell Infiltration

The Cell-type Identification by Estimating Relative Subsets of RNA Transcripts (CIBERSORT) algorithm evaluated the correlation between the cDC1RS and 22 immune cell subtypes, based on their expression profiles. Analyses included gene markers for various immune cell types, such as T cells, CD8+ T cells, B cells, monocytes, tumor-associated macrophages (TAMs), M1 and M2 macrophages, dendritic cells (DCs), neutrophils, NK cells, and various T helper and regulatory cell subtypes.

### 2.3. Identification of Oncogenic Signaling Pathways

Gene set variation analysis (GSVA) was employed to estimate each gene set's enrichment score, based on gene expression levels within individual samples from the TCGA-STAD cohort. This analysis encompassed several oncogenic signaling pathways, including RTK-RAS, Notch, Hippo, *β*-catenin/Wnt, PI-3-Kinase/Akt, cell cycle, TGF*β*, Myc, and P53.

### 2.4. Statistical Analysis

R software and SPSS were utilized for statistical analyses. Continuous variables were compared between two groups using the *t*-test, while categorical variables were assessed through chi-square or Fisher's exact test, as appropriate. Spearman's correlation coefficient was applied for bivariate quantitative analyses. Kaplan–Meier survival curves, complemented by log-rank tests, were used to analyze survival data. Hazard ratios and 95% confidence intervals were calculated using the Cox proportional hazards model. Statistical significance was set at *p*  < 0.05, with varying levels of significance indicated by  ^*∗*^*p*  < 0.05,  ^*∗∗*^*p*  < 0.01, and  ^*∗∗∗*^*p* < 0.001.

## 3. Results

### 3.1. Evaluation of the cDC1-Related Gene Associated Subgroups with Prognosis in Gastric Cancer

**Figure 1:**
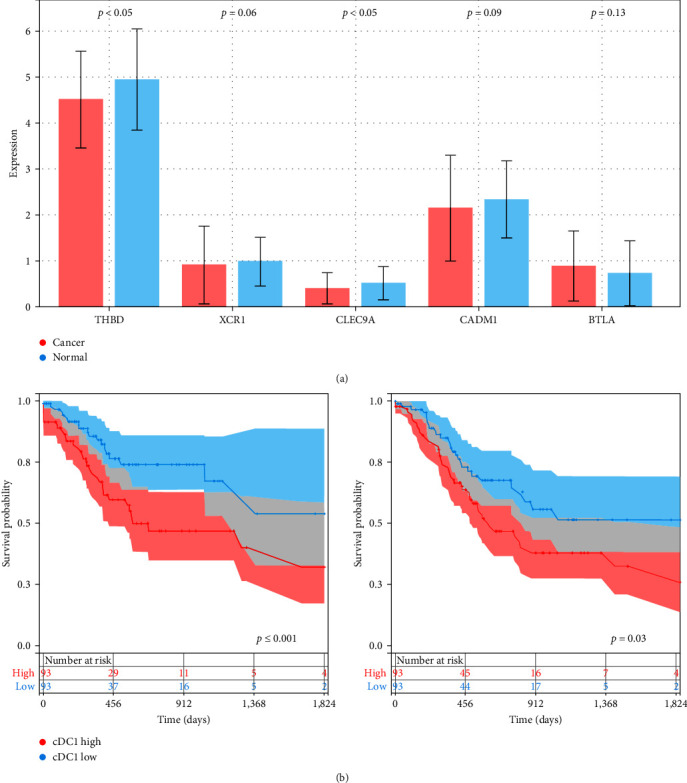
Association of cDC1-related gene expression with survival outcomes. (a) The box plot displays the distribution of average expression for genes THBD, XCR1, CLEC9A, CADM1, and BTLA derived from TCGA samples. (b) Kaplan–Meier plots highlight the relationship between the cDC1-related signature and both overall survival duration and disease-free survival period.

We first identified the most common five cDC1-associated genes such as THBD (CD141), XCR1, CLEC9A, CADM1, and BTLA with literature searches. We examined the differential expression of cDC1-related genes between the tumors and normal tissues from the TCGA-STAD dataset. Notably, while THBD had the highest expression levels among five genes, CLEC9A showed the lowest expression compared with normal samples (Figure 1(a)). To evaluate the prognostic impact of conventional type 1 DC-related gene signature, we performed survival analysis between cDC1-high and cDC1-low groups using the TCGA cohort. It revealed that cDC1-high group was associated with poor overall survival (*p* < 0.001) and disease-free survival (*p*=0.03) (Figure 1(b)).

We explored the association between the cDC1RS and clinical features (Table 1). The cDC1-high group was significantly associated with the advanced T stage (*p*=0.02) and high histologic grade (*p* < 0.001). The low group was associated with MMR-deficient status (*p* < 0.001).

Next, we further performed a univariate and multivariate Cox regression analysis to elucidate the independent predictive role of cDC1RS in GC. High cDC1-related gene expression predicted significantly poor patient prognosis (HR (95% CI), 1.834 (1.114–3.020); *p*=0.017) than low expression group in GC (Table 2). These results suggest that the cDC1-high expression has good robustness for predicting prognosis of GC.

### 3.2. The Association of DC Function and Microenvironment Landscape with Expression of cDC1-Related Genes in Gastric Cancer

**Figure 2:**
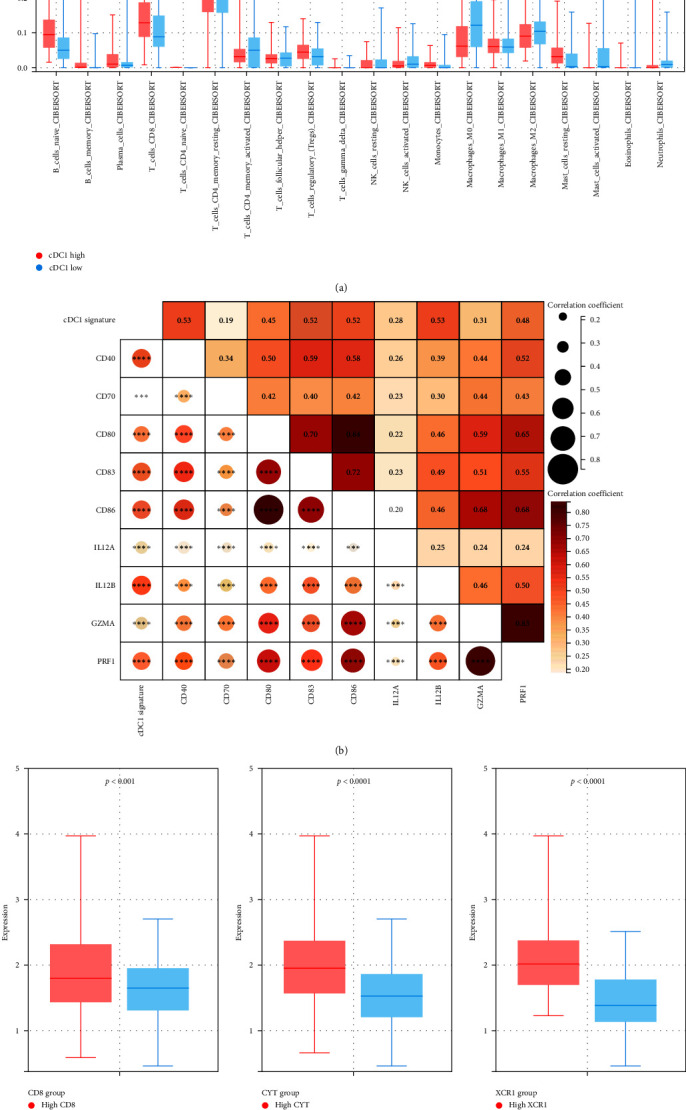
Biological traits based on the categorization of the cDC1-related signature. (a) CIBERSORT analysis indicating immune cell infiltration levels in cDC1-high and cDC1-low groups. (b) Heatmap illustrating gene expression relationships with costimulatory molecules, IL-12 activity, and cytotoxic functionality. (c) Box plot depicting variations in the cDC1-related signature across groups defined by CD8+ T cell presence, cytotoxic action, and XCR1 expression.

Our study focused on elucidating the role of conventional type 1 dendritic cell-related signatures (cDC1RS) in gastric cancer (GC), particularly in relation to the tumor microenvironment and dendritic cell functionality. We utilized the CIBERSORT algorithm to assess immune cell infiltration differences between cDC1-high and cDC1-low expression groups in GC. The cDC1-high group exhibited considerably elevated expression of CD8+ T cell (*p* < 0.01) as well as T regulatory (Treg) cells (*p* < 0.01) (Figure 2(a)).

The interaction of mature cDC1s with T cells is crucial for the proliferation of CD8+ T cells and the initiation of effective antitumoral responses. This study thoroughly examined the relationship between cDC1RS and markers of mature cDC1, including CD40, CD70, CD80, CD83, and CD86. These costimulatory molecules play a pivotal role in the activation of cDC1 following antigenic signal transmission to T cells. However, inadequate stimulus transmission can induce T cell anergy. Our results revealed a significant positive correlation between cDC1RS and all costimulatory molecule markers (Figure 2(b)). Cytokine signaling, essential for effective T cell polarization, was also evaluated. The IL-12 is responsible for proliferation and differentiation of CD4+ T cells into effector T cells. Thus, we explored the connection between cDC1-related gene expression and IL-12A and IL-12B. The increased expression of IL-12A and IL-12B was significant associated with higher expression of cDC1RS (Figure 2(b)). Furthermore, we investigated the relationship between cDC1RS and the expression of GZMA and PRF1, markers indicative of CD8+ T cell cytotoxic activation (Figure 2(b)). Both were positively correlated with cDC1RS.

In groups classified by CD8+ T cell infiltration and cytotoxicity, the cDC1RS was notably elevated in those with high CD8+ T cell and high cytotoxicity groups (Figure 2(c)). XCR1, which marks the terminal differentiation of human cDC1, is associated with their comprehensive range of effector functions. Intriguingly, group with high XCR1 expression also displayed a pronounced expression of the cDC1RS (Figure 2(c)).

Our findings suggest there was a strong association between cDC1RS and CD4 and CD8+ T cell-mediated immune responses, potentially leading to more effective T-cell priming in the tumor microenvironment. These consequences suggested that patients in cDC1-high group had a higher potential to benefit from immunotherapy.

### 3.3. Mechanism of Immune Escape in cDC1-Related Signature

To better explore the potential mechanism for the paradoxical prognosis conflicting features of tumor microenvironment of the cDC1-high group, we further evaluated which pathways or mechanism were contributed to immune evasion of GC.

**Figure 3:**
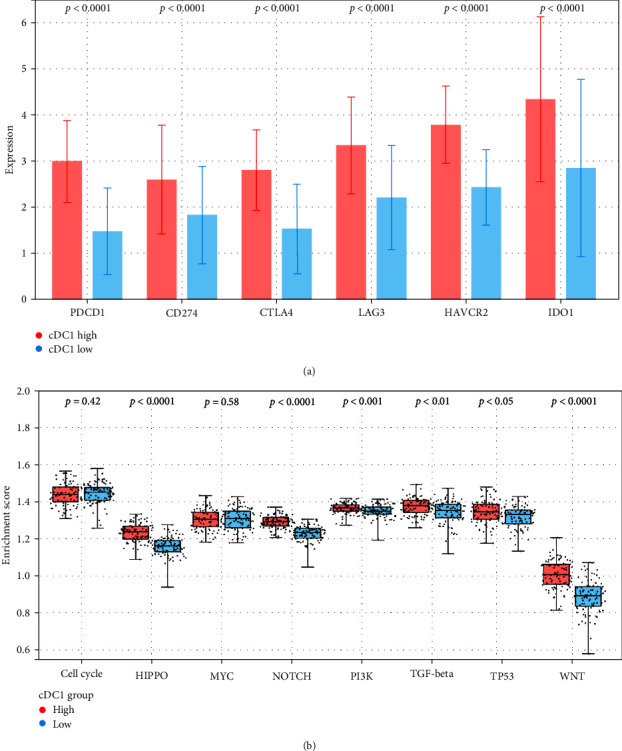
Comparative analysis of transcriptional expression of ICP genes and oncogenic signaling pathways in cDC1 low vs. high groups. (a) Comparative analysis of transcriptional expression levels of ICP genes in cDC1-low and cDC1-high groups. (b) Differences in the oncogenic signaling pathway between cDC1 low and high groups.

The immune system has different negative feedback loops to shut down an immune response, which can be hijacked by the tumor to protect itself. Cancer cells upregulate not only PD1 (PDCD1) but also other immune check points (ICPs) such as PD-L1 (CD274), cytotoxic T-lymphocyte-associated protein 4 (CTLA-4), lymphocyte-activation gene (LAG)-3, hepatitis A virus cellular receptor 2 (HAVCR2), and indoleamine 2,3-dioxygenase 1 (IDO1), which, upon ligand triggering, reduce (some of) CD8+ T cell effector functions. We compared the expression profiles of immune check points between the cDC1-high and cDC1-low groups (Figure 3(a)). The expression of 6 ICPs genes such as PDCD1, CD274, CTLA-4, LAG-3, HAVCR2, and IDO1 were significantly upregulated in cDC1-high group compared with the cDC1-low group.

Among the different CD4+ T cell subpopulations, there are Treg characterized by the expression of the transcription factor FoxP3. Treg can suppress CD8+ effector cells both in an antigen-specific and antigenunspecific way. As shown in Figure 2 (a), the infiltration of Treg cells is high in cDC1-high group.

Next, TCGA data have confirmed the roles of canonical oncogenic signaling pathways as influencing cancer progression—cell cycle, Hippo, Myc, Notch, PI3K, TGF*β*, P53, and *β*-catenin/Wnt (Wnt) [14]. We used GSVA to compare with enrichment score (ES) of oncogenic signaling pathways across cDC1 groups to identify significant differences in their activities in the different subtypes. Based on GSVA analysis (Figure 3(b)), we observed that the ESs of the Hippo, Notch, PI3K, TGF*β*, Tp53, and WNT pathways were high score in cDC1-high group. Among them, the TGF*β* pathway ES was the highest in cDC1-high group, and the Wnt pathway ES was the largest difference between two groups. It is well-known that the Wnt and TGF-*β* pathways instruct tolerance and suppress immune reaction [15] in cancer.

Taken together these results, to suppress the effector functions of CD8+ T cells, GCs with high cDC1 evolve into cancer cells with enhanced suppressive functions, such as upregulation of ICP and oncogenic pathways responsible for tolerance maintenance and immune evasion, which are likely used by tumor cells to promote survival.

### 3.4. Clinical Relevance of cDC1-Related Signature in GC Treated with Chemotherapy and Immunotherapy

**Figure 4:**
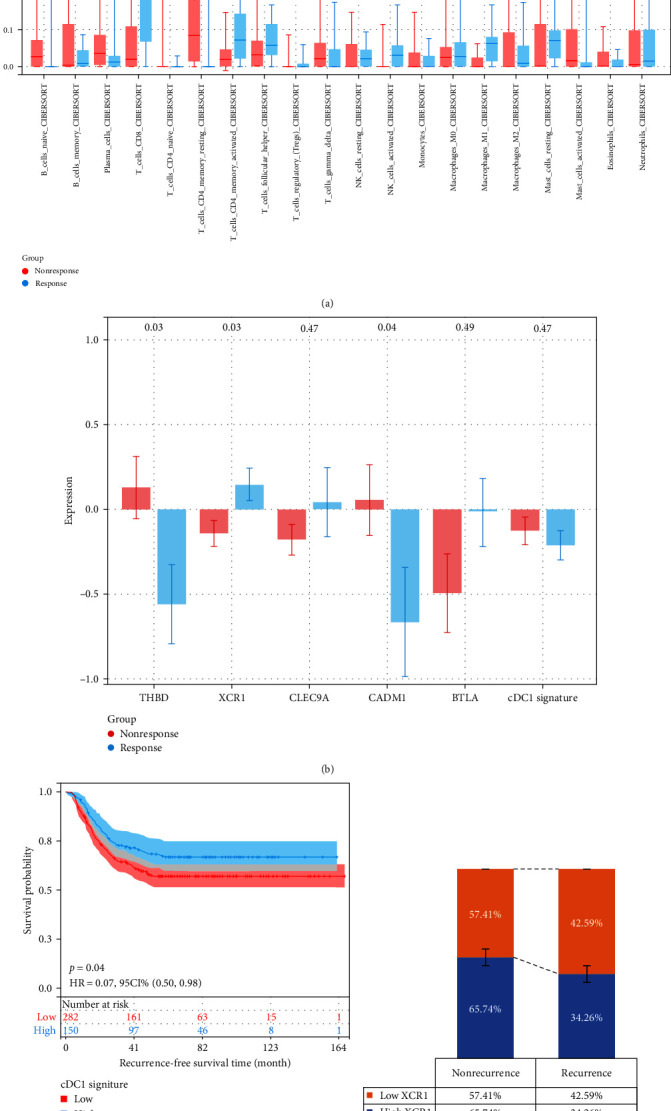
The assessment of therapeutic efficacy in cDC1-related signature. (a) Distribution of tumor-infiltrating immune cells in immunotherapy responder versus nonresponder groups. (b) Comparative expression of cDC1-associated genes in immunotherapy responders and nonresponders. (c) Influence of the cDC1-associated signature on disease-free survival among GC patients with or without chemotherapy history. (d) Relationship between XCR1 expression levels (low vs. high) and recurrence status (nonrecurrence vs. recurrence).

Based on the findings revealing the significance of cDC1RS at the transcript level, we asked whether cDC1RS can be used as a predictor for ICI response. We estimated the relative fraction of 22 immune cells for each patient between in the responders and nonresponders groups by using CIBERSORT using RNA-seq data from the PRJEB25780 dataset. Wherein CD8+ T cells, activated NK cells, and macrophage M1 demonstrated the most significant higher infiltration in response group, Tregs were not statistically different (Figure 4(a)). Subsequently, we aimed to assess the impact of cDC1-associated genes on advanced gastric cancer patients treated with ICIs. We examined bulk RNA-seq data from the PRJEB25780 dataset, which assessed the efficacy of pembrolizumab in a subset of metastatic GC patients. In Figure 4(b), elevated XCR1 expression was associated with complete or partial responses (CR/PR), although the other genes did not.

Next, we assessed the impact of the cDC1RS on advanced gastric cancer patients who underwent curative surgery complemented with adjuvant chemotherapy, utilizing the GSE26253 dataset. As of the data cutoff point, none of the study participants had been treated with ICIs. Interestingly, patients with a high cDC1 signature demonstrated improved overall survival (HR 0.70, *p*=0.04) (Figure 4(c)). We delved deeper to determine if the expression of the XCR1 gene varied depending on the occurrence or nonoccurrence of recurrence, as previously illustrated. As shown in the Figure 4(d), patients with higher expression levels of XCR1 have marginally higher nonrecurrence rates and conversely lower recurrence rates as compared to those with lower levels of expression.

In sum, the findings indicate that the cDC1 signature holds potential in identifying patients poised to respond favorably to ICIs or chemotherapy, established treatments for advanced gastric cancer.

## 4. Discussion

In the present study, we delved into the relationship between the cDC1-associated signature and the tumor microenvironment in GC, harnessing gene expression profiles to devise a prognostic biomarker elucidating the molecular interactions related to patient outcomes. While the prognosis appeared poorer in the cDC1-high group compared to the cDC1-low group, our findings revealed a positive correlation between the expression of the cDC1RS and both the maturation of cDC1 and the proliferative and cytotoxic capacities of CD8+ T cells. Additionally, our research suggests that XCR1 expression may serve as an indicator of mature, cytokine-secreting cDC1 that stimulates protumoral immunity. We propose that the cDC1RS could be indicative of prognosis and predictive of the response to therapy such as ICIs and chemotherapy, potentially due to a synergistic interplay or cross-communication between cDC1 and CD8+ T cells in the context of GC.

Conventional type 1 dendritic cells (cDC1) are known for their exceptional ability to cross-present antigens, leading to the activation of cytotoxic CD8+ T cells, crucial for immune responses [13, 16]. Notably, CD8+ T cells rapidly initiate a robust immune response upon antigen recognition, primarily via potent interaction with cDC1. Dorner et al. [17] provided evidence that cDC1, characterized by XCR1 expression, instigates increased CD8+ T cell proliferation and promote interferon-gamma (IFN*γ*) secretion. However, the role of this dynamic in solid tumors, especially gastric cancer (GC), remains insufficiently explored.

The prognostic implications of cDC1 infiltration have been studied in various epithelial cancers and present conflicting results with a number of studies reporting both favorable [6, 18, 19, 20] and unfavorable outcomes [21, 22]. In cancers such as melanoma, breast, head and neck, and lung, a subset of research indicates improved patient survival outcomes, crediting this to efficient antigen presentation, bolstered cytotoxic CD8+ T cell activity, enhanced NK cell recruitment, and a dominant IFN-*γ* response [18, 19, 20]. Conversely, the complexity of the tumor microenvironment can sometimes render cDC1s less effective or even associate with adverse outcomes, as evidenced in certain breast cancer subtypes, where the presence of cDC1s in an lymphocyte-rich or lymphocyte-depleted context correlates with reduced survival, hinting at an immune contexture-dependent role [22]. Understanding these aspects could offer valuable insights into the complex role of cDC1s in tumor immunity and potentially guide more targeted therapeutic strategies. Such an investigation could illuminate whether the variable outcomes of cDC1 infiltration are due to differing microenvironmental pressures or distinct tumor–immune interactions, thereby providing a more nuanced understanding of cDC1′s role in cancer progression and treatment response.

In gastric cancer, the prognostic implications of cDC1 remain under-investigated. We first noted reduced expression of some cDC1-associated genes in GC tumor tissues. The CD141 (THBD and CLEC9A genes, along with XCR1, CADM1, and BTLA, play a vital role in promoting antitumor immunity through aiding in antigen presentation and T cell activation. Their reduced expression in tumors indicates a tumor-driven suppression of immune function. Based on these findings, we hypothesize that this reflects the complexity of the TME and the tumor's immune evasion mechanisms. And we think it is crucial to comprehend how alterations in cDC1-related gene expression impact the immunological status and, subsequently, the clinical prognosis of GC. Next, we discovered that a heightened cDC1RS correlates with reduced overall and disease-free survival within the TCGA-STAD cohort, suggesting these genes' potential prognostic relevance in GC. Despite no direct survival benefit correlation, we observed that a marked variation in CD8+ T cell prevalence was noted between high and low cDC1RS groups, pointing to significant microenvironmental remodeling. Our analyses of costimulatory molecule markers and cytokine secretion related to cDC1s revealed a strong association between the cDC1RS and indicators of T lymphocyte cytotoxicity and dendritic cell activation. These results suggest that elevated cDC1RS levels in GC are linked to standard cDC1 maturation processes, fostering an antitumor immune microenvironment through the activation and integration of endogenous T cells. This insight underlines the importance of cDC1s in shaping GC's immunological landscape, with significant implications for future therapeutic interventions.

The detection of T cells within the tumor microenvironment suggests the presence of escape strategies that permit the simultaneous existence of antitumor immune responses alongside the tumor [23]. A notable observation is the correlation between CD8+ T cell infiltration and the activation of immunosuppressive pathways. This includes the recruitment of Tregs, upregulation of ICPs inhibiting T cell activity, and the secretion of suppressive cytokines such as TGF-*β*, a factor commonly observed in various studies [15, 24, 25]. Tregs, recognized for their immunosuppressive functions, adopt various strategies like secretion of inhibitory molecules, metabolic disruption, and suppression of dendritic cell functions [26]. The initial discovery of Treg elevation in cancer patients by June et al. [27] underscored their potential role in tumor progression. The prognostic role of Tregs in GC is subject to debate, with several studies identifying them as a negative prognostic indicator [28, 29], while others have reported the opposite [30, 31]. More importantly, the current research indicates that Tregs within the tumor microenvironment are complexly regulated and may influence the course of GC through their stability and plasticity [32, 33, 34]. In addition, TGF-*β* signaling critically moderates immune tolerance and inflammation, influencing the function of various immune cells, including the suppression of dendritic cell antigen presentation and promotion of a Treg-favorable, tolerogenic environment [15, 35, 36, 37]. Additionally, the Wnt pathway is recognized for its regulation of T-cell development and activation, as well as dendritic cell maturation [38]. Dysfunctional TGF-*β* or WNT signaling is a common feature in numerous tumors such as gastric cancer, often with concurrent alterations in both pathways [39, 40, 41]. ICP expression on tumor cells, a well-known mechanism facilitating tumor evasion from T cell-mediated responses, has been linked with GC prognosis [25, 42]. Kim et. al have shown that immune checkpoints, such as CTLA-4 and PD-1, when expressed, were correlated with survival rates in patients with resectable GC [43], and various other with PD-L1 (+) expression have been reported to have poor prognoses [44, 45]. In our study, the high cDC1RS was characterized by increased Treg and elevated ICPs alongside intensified TGF-*β* and WNT signaling activity, suggesting an immunosuppressive milieu. This complex regulatory environment could be pivotal in shaping the immunosuppressive landscape, thereby influencing the prognosis for GC patients.

Treatment response is a primary prerequisite for improving cancer survival. Currently, surgical resection and adjuvant chemotherapy remain the primary approaches for advanced-stage GC management [46]. The recent the Food and Drug Administration approval of pembrolizumab, in combination with trastuzumab and chemotherapy for HER2-positive advanced gastric cancer (AGC), signifies a shift toward more targeted treatment modalities [47]. The focus of our current study is to identify reliable prognostic and predictive, aiding in the development of personalized treatment regimens for GC patients.

In the realm of immunotherapy and chemotherapy, the presence or absence of tumor-specific T cells marks a significant distinction between responsive and nonresponsive cancer patients [48, 49, 50]. Tumeh et al. [48] provided evidence that the presence of CD8+ T cells within the tumor or at the invasive margin is highly correlated with response to PD-1 inhibition. Wang et al. [50] demonstrated that intratumoral CXCR5+CD8+T cells are associated with better overall survival in gastric cancer patients and benefit from adjuvant chemotherapy.

Thus, we hypothesize that the upregulation of immunomodulatory molecules in the cDC1-high group might reflect an induced immune activation driven by effector immune cells, and despite their upregulation as a negative feedback to dampen antitumor immunity, the activated immune system could still attack the tumor, offering a therapeutic advantage over unactivated immunity. Gene screening related to immunotherapy and chemotherapy responses revealed a notable association between high XCR1 expression and enhanced immunotherapy response. Furthermore, GC patients with a high cDC1 signature exhibited favorable outcomes in the adjuvant chemotherapy group, with XCR1 expression also being predominant in the nonrecurrent cohort. These findings suggest the potential of cDC1RS and XCR1 as biomarkers for stratifying GC patients into low-risk and high-risk categories based on their sensitivity to immunotherapy and chemotherapy.

However, there are some limitations in the present research. Firstly, our analysis was mainly based on online databases. The role of five genes in GC was less analyzed, and their specific molecular mechanisms require further in vivo/in vitro assays. Secondly, the cDC1-based signature should be verified by prospective, large-scale cohorts before clinical application. Moreover, larger clinical trials are needed to validate the role of the cDC1 signature in antitumor drug selection.

## 5. Conclusion

Although the unique role of conventional type 1 dendritic cells (cDC1s) in immune control has been widely confirmed [11], few studies have compared immune cell infiltration, gene expression, and prognostic impact according to the cDC1 signature in cancer such as gastric cancer. Through cDC1-related genes, we provide valuable insights into the complex and paradoxical tumor microenvironment formation in GC. Additionally, these cDC1RSs may offer valuable information for prognostic stratification. This study may contribute to developing novel therapeutic strategies for STAD patients and improving their long-term prognoses.

## Figures and Tables

**Table 1:** Clinicopathological characteristics according C1DC expression in GC.

Characteristics	High group (*n*, %)	Low group (*n*, %)	*p* Value
C1DC signature expression			
Median (range)	2.40 (2.15−3.97)	1.13 (0.47−1.33)	*—*
Age (year)			0.24
<65	49	40	—
≥65	44	53	—
Sex			0.55
Female	33	38	—
Male	60	55	—
pT category			0.02
pT1	0	9	—
pT2	18	20	—
pT3	43	43	—
pT4	28	21	—
Node metastasis			0.26
Absent	21	29	—
Present	67	60	—
Distant metastasis			0.95
Absent	79	83	—
Present	7	6	—
Prognostic stage			0.45
I–II	30	33	—
III–IV	47	38	—
Grade			<0.001
G1	0	3	—
G2	24	46	—
G3	66	43	—
MMR status			<0.001
MMR deficient	2	22	—
MMR proficient	84	61	—

MMR, mismatch repair.

**Table 2:** Univariate and multivariate analyses of overall survival in GC.

Variable	Category	Univariate analysis		Multivariate analysis	
		HR (95% CI)	*p* Value	HR (95% CI)	*p* Value
Age	≥65 vs. <65 years	1.580 (1.122−2.224)	0.008	2.291 (1.386−3.167)	0.001
Sex	Male vs. female	1.200 (0.844−1.707)	0.308	—	—
pT stage	T1–2 vs. T3–4	1.810 (1.178−2.781)	0.007	1.822 (0.963−3.445)	0.065
N stage	N1–N3 vs. N0	1.803 (1.197−2.718)	0.005	1.453 (0.738−2.858)	0.927
M stage	M1 vs. M0	2.338 (1.343−4.068)	0.003	1.787 (0.715−4.463)	0.214
C1DC group	High vs. low	1.683 (1.053−2.690)	0.028	1.834 (1.114−3.020)	0.017
Histologic grade	G3 vs. G1–2	1.366 (0.963−1.937)	0.081	—	—
MMR status	Deficient vs. proficient	0.706 (0.438−1.138)	0.152	—	—

HR, hazard ratio; CI, confidence interval; MMR, mismatch repair.

## Data Availability

The datasets presented in this study can be found in online repositories. The names of the repository/repositories and accession number(s) can be found in the article.
